# Developing Interventional Radiology Anticoagulation Guidelines: Process and Benefits †

**DOI:** 10.3390/jcm7040085

**Published:** 2018-04-20

**Authors:** J. Scott Kriegshauser, Howard H. Osborn, Sailen G. Naidu, Eric A. Huettl, Maitray D. Patel

**Affiliations:** 1 Department of Radiology, Mayo Clinic Hospital, 5777 E Mayo Blvd, Phoenix, AZ 85054, USA; naidu.sailen@mayo.edu (S.G.N.); Huettl.Eric@mayo.edu (E.A.H.); Patel.Maitray@mayo.edu (M.D.P.); 2 Department of Radiology, Mayo Clinic, Scottsdale, AZ 85259, USA; Osborn.Howard@mayo.edu

**Keywords:** anticoagulation, guidelines, interventional, safety

## Abstract

We created, posted, and updated radiology department anticoagulation guidelines and identified various steps in the process, including triggering events, consensus building, legal analysis, education, and distribution of the guidelines to nurses and clinicians. Supporting data collected retrospectively, before and after implementation, included nursing satisfaction survey results and the number of procedure cancellations. After the guidelines were developed and posted, significantly fewer procedures were cancelled, nursing satisfaction was higher, and radiologists performed procedures with less variability. Anecdotally, radiologists had fewer queries about anticoagulation. The development and dissemination of radiologic procedure anticoagulation guidelines should be considered as a departmental quality improvement project.

## 1. Introduction

In our institution’s Department of Radiology, different opinions on preprocedure anticoagulation issues led to confusion and delays in ordering and scheduling procedures. Opinions about best practices varied among radiologists between divisions and within divisions because of individual practice habits and unfamiliarity with the relevant available evidence. A procedure approved by one radiologist might be assigned to another radiologist who was not comfortable with the previous approval. This led to instances of clinicians searching for a radiologist who was willing to do a procedure on an anticoagulated patient. New anticoagulants and antiplatelet medications made decisions even more difficult, and having to address anticoagulation questions decreased the efficiency of the radiologists.

Initial attempts to standardize practice in the Department occurred at the level of the divisions. The Ultrasound (US) Division was the first to institute basic guidelines. Before 2008, that group had established an informal guideline stating that paracentesis and thoracentesis could be performed on anticoagulated patients. Subsequently, according to evidence from Atwell et al. [1], members of the US Division decided, as a group, that holding aspirin for five days was preferred, but not required, for US-guided procedures. Nurses calling patients before a procedure could proceed without notifying the radiologist even if aspirin had not been held for five days. This differed from the practice in other divisions, confusing nurses, and leading to frustration and tension among team members. 

**Figure 1:**
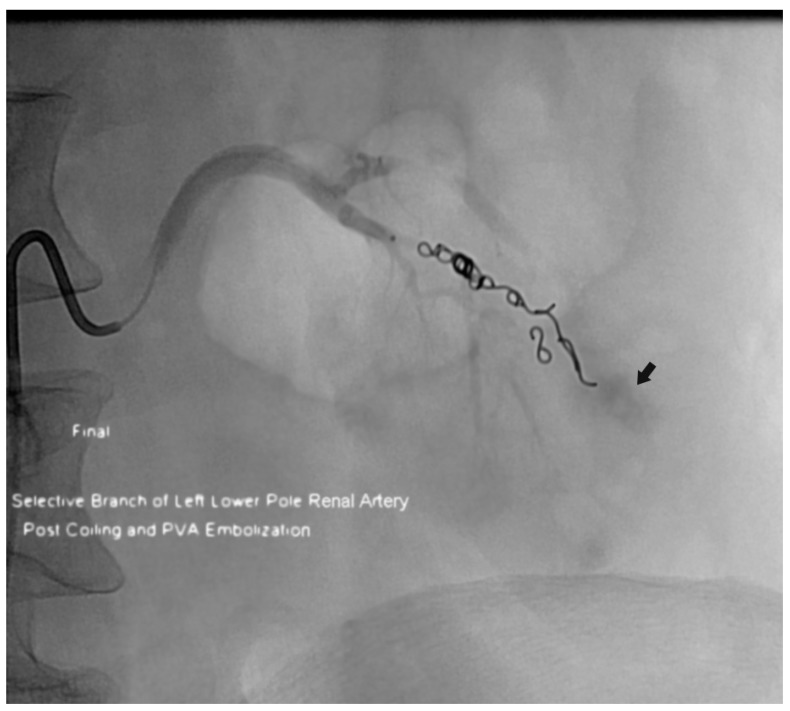
Patient who had active bleeding after an ultrasonographically-guided left native kidney biopsy. This image is from the postprocedure arteriogram after coil and particle embolization of a lower pole renal artery branch. Residual extravasated contrast material is still evident (arrow). The patient was receiving a subcutaneous low-molecular-weight heparin bridge, with the last dose given approximately 6 h before the procedure.

In the summer of 2012, a cluster of four adverse postprocedure bleeding events were reported during one month (Figure 1). Before this, it was very unusual for the Department of Radiology to have more than one procedure-related bleeding event reported in a month. The effect of preprocedure anticoagulation issues for two of the patients was unclear, and this heightened the sense of urgency that a uniform department-wide approach was needed. As a result, a task force was formed to develop consensus and to create guidelines for improving patient safety and streamlining the clinical process.

## 2. Materials and Methods

Institutional review board approval was not required for this study in accordance with the Code of Federal Regulations, 45 CFR part 46. The timeline of events is summarized in Table 1.

### 2.1. Anticoagulation Task Force

The Anticoagulation Task Force was formed to create anticoagulation guidelines. The chair was a physician member of the US and Interventional Radiology (IR) Divisions. Other members were the Radiology nurse supervisor (and quality specialist), IR nursing team lead, Radiology nursing team lead, physician–head of the US Division, physician–head of the IR Division, and a physician who was a member of the IR Division and head of departmental quality oversight. The charge of the task force was to form consensus among the radiologists performing procedures and, to the extent possible, generate specific guidelines for nurses and ordering clinicians. Radiologically-guided procedures are performed by radiologists in multiple divisions of the Department of Radiology, including IR, US, Musculoskeletal, Neuroradiology, and Breast. The IR Division handles all computed tomographically (CT)-guided body and chest biopsy procedures. 

The initial work for the task force was to (1) create a list of anticoagulation medications (Table 2); (2) review manufacturer data for half-lives, monitoring methods, and guidelines; (3) review the literature, including Society of Interventional Radiology (SIR) guidelines [1,2,3,4,5,6,7,8]; (4) review available guidelines from other institutions; and (5) create the first draft of the departmental guidelines. At the beginning of the consensus process, the chair of each division was given the initial version of the guidelines and radiologists were given the appropriate drug information and literature results. The first set of approved guidelines included critical laboratory test results (if the international normalized ratio >1.5 or if the platelet count <50 × 10^9^/L, notify the radiologist) and initial restart guidelines (restart low-molecular-weight heparin, if needed, 48–72 h postprocedure, and resume all anticoagulation medications the next day unless otherwise specified by the ordering provider).

### 2.2. Implementation

The guidelines were provided to all nurses and radiologists who were involved with procedures. Nurses contacted radiologists or ordering clinicians only for variances.

We reviewed and updated our guidelines, when applicable, as described in an article by Baron et al. [9]. This article and the SIR guidelines were instrumental in receiving final approval from the Legal Department and institutional committees [7]. We also expanded our postprocedure restart guidelines with recommended times to resume the dose for each medication and added new medications (Table 2 and Table 3). This remains an ongoing process, including a review of new and updated literature [10,11,12,13].

After we received approval from various committees and our institution’s legal department, the guidelines were finalized (Table 3). The document was posted on the intranet, which can be accessed through our Department of Radiology website. Ordering clinicians could refer to the guidelines before ordering a procedure and discuss with the patient the timing and any changes or adjustments of anticoagulation medications. All nurses in the institution had access to the information.

### 2.3. Metrics

We compared results from departmental nursing satisfaction surveys that were performed in 2011 and 2015 (survey responses were based on a five-point scale). The total number of procedures ordered with US or CT guidance and the number subsequently cancelled were recorded and compared for 2012 (before use of the guidelines) and 2015 (the first complete year after institutional use of the guidelines started in March 2014). The reasons for cancelling examinations were not documented. Adverse events were continually tracked through the same period. The degree of consensus among the performing radiologists was recorded.

## 3. Results

Overall nursing satisfaction (percentage responding “mostly satisfied” or “very satisfied”) within our Department of Radiology increased from 71% in 2011 to 88% in 2015. Favorable responses (“mostly agree” and “agree” responses) increased between 2011 and 2015 as follows:

1. From 64% to 94% for the statement, “[My institution] is committed to a culture of safety.”
2. From 21% to 50% for the statement, “I feel free to speak my mind without fear.”
3. From 64% to 67% for the statement, “I am satisfied with my involvement in the decisions that affect my work.”

In 2012, a total of 1192 US procedures were ordered, with 914 (77%) completed and 278 (23%) cancelled. In 2015, a total of 1397 US procedures were ordered, with 1129 (81%) completed and 268 (19%) cancelled. The percentage of US procedure cancellations was significantly less in 2015 than in 2012 (*p* < 0.001; χ^2^ test). In 2012, a total of 687 CT procedures were ordered, with 489 (71%) completed and 198 (29%) cancelled. In 2015, a total of 666 CT procedures were ordered, with 529 (79%) completed and 137 (21%) cancelled. The percentage of CT procedures cancellations was significantly less in 2015 than in 2012 (*p* = 0.02; χ^2^ test). Cancellations for both US and CT (combined) decreased significantly between 2012 and 2015 (*p* < 0.001; χ^2^ test). 

Consensus was achieved among the radiologists performing procedures, with only one exception in the performance of lung nodule biopsies: two of five radiologists who perform lung nodule biopsies still hold aspirin for five days before performing the procedure. Except in this circumstance a clinician receives the same recommendation from any radiologist. All radiologists held aspirin for five days (consensus was achieved) before performing stereotactic or magnetic resonance imaging–guided breast biopsies. For these procedures, nurses continued to discuss variances with the radiologists involved. 

Two adverse events were reported during the next 30 months.

## 4. Discussion

The underlying issues we encountered in this process are likely prevalent in the radiology community. For departments that have yet to tackle the process of developing guidelines, forming consensus, and making the final result easily accessible, we hope that our experience will serve as a guide or a starting point for discussion. The potential benefits include increased quality, safety, efficiency, and job satisfaction for all involved. 

Medicolegal implications arise from the development and use of clinical practice guidelines. Obtaining legal review during development was important and helpful. For physicians and other health care providers, practice guidelines serve as recommendations and are not intended as actual standards of care. However, for attorneys handling medical malpractice claims, practice guidelines are sought and used as evidence of the legal standard of care—that is, what a reasonable provider should do in similar circumstances. When an adverse event occurs, failure to meet an institutional guideline creates potential liability for a provider at that institution. 

Practice guidelines should be formally reviewed and adopted by an institution, with appropriate communication to relevant providers before the effective dates of the guidelines. The guidelines should include a statement that they are intended as recommendations and not actual standards of medical care. The guidelines should be dated and updated to stay current with advances in the practice. The guidelines may include instructions on what to do if the practice guidelines cannot be met, such as delaying or rescheduling the procedure. 

We had only a limited amount of measurable data available to us, largely because we did not treat this process as a formal quality improvement project from the start. Much of our perceived benefit is anecdotal, although it is supported by the measures of increased nurse satisfaction, decreased procedure cancellations, and nearly complete consensus among performing radiologists. Overall nursing satisfaction was affected by other changes in the work environment (e.g., feeling more free to speak up without fear), which is a limitation of the data. Still, the strong increase (from 64% to 94%) in agreement that the institution was committed to a culture of safety is good evidence that developing the guidelines helped to increase overall satisfaction. The procedure cancellation data may have been affected by other factors, which is also a limitation. If other departments undertake this process, we strongly encourage them to set up a formal quality improvement project and collect data before and after the new process is implemented. 

The number of bleeding complications (two occurrences) for the next 30 months was less than in the preceding period—a cluster of five events prompted this process—although it may have been influenced by other random factors. Certainly one could argue that the data so far imply a positive effect, although more time and more comparison data would be required for statistical confirmation.

An Internet search showed that several institutions have posted anticoagulation guidelines for radiology procedures. These vary in content and extent and are publicly available. The risk of posting guidelines outside an intranet firewall is controversial, and if an institution wants to post guidelines that are publicly available, we recommend additional consultation with the institution’s legal department beyond that obtained for guidelines posted internally. The wording is important, including an explanation that they are only guidelines, similar to the SIR guidelines [7,10]. Published references are also important for validation.

We have provided recommendations on when to resume medications, which are not included in the current SIR guidelines, although they are available to a limited extent in other references [4,7,9,10,13]. This would be a prime area for future research using prospective trials. Questions on resuming medications are common and time-consuming, but the use of many medications requires a discussion with providers to decide on a course of action for each patient. Further investigation is ongoing to correlate specific anticoagulants with specific procedure-related risk along with our continuous updates to the guidelines based on changes in available medications. The recent US Food and Drug Administration approval of idarucizumab, a reversal agent for dabigatran, may also influence how we manage medications.

Obtaining consensus can be difficult, especially when several different expert opinions exist. For example, Baron et al. [9] recommend holding dabigatran 1 to 2 days if creatinine clearance (CrCl) is 50 mL/min or more and for 3 to 5 days if CrCl is less than 50 mL/min. Patel et al. [10] recommend holding 2 to 3 days if CrCl is 50 mL/min or more and 3 to 5 days if CrCl is less than 50 mL/min. Douketis et al. [4] recommend holding for five days. The SIR consensus guidelines reported by Patel et al. [7] reflected an 80% consensus for all but a few of the recommendations. Within a single department, this will be easier than trying to develop universal guidelines. In this era of computer order entry, we are developing a direct link to our guidelines when a referring clinician places any relevant order. This will provide prompts on how to proceed when a patient is anticoagulated or needs to receive bridging therapy. 

Limitations of this report have been discussed above for the data measured. We reiterate the importance of collecting data before and after the process is implemented for a quality improvement project, and we realize that this is the greatest limitation of this report.

In conclusion, we present the processes that may be involved when developing and posting departmental preprocedure and postprocedure anticoagulation guidelines. We stress the importance of using a departmental, rather than divisional, approach, obtaining as much consensus as possible, involving legal counsel, and actively updating the guidelines. In our experience, increased nursing satisfaction and improved efficiency (i.e., fewer procedure cancellations) are the primary benefits of having department guidelines that are widely available within an institution. We have also reported a decrease in procedure-related bleeding events, although the relationship of this development to the existence of our guidelines is not clearly established. There are many other perceived benefits, as discussed above, which we hope other departments will measure as part of a formal quality improvement project.

## Figures and Tables

**Table 1:** Timeline of events in the Department of Radiology.

Date	Event
Before June 2012	Ultrasound Division guidelines for allowing paracentesis and thoracentesis on anticoagulated patients, with a preference (not a requirement) for holding aspirin for biopsies
June and July 2012	Five procedure-related bleeding complications occurred
August 2012	Anticoagulation Task Force was formed to develop departmental anticoagulation guidelines
October 2012	Anticoagulation Task Force created first draft of guidelines
January 2013	(1) Consensus on the guidelines was reached in all divisions of the Department
	(2) Institution’s Legal Department gave its first opinion on the guidelines with specific suggestions
	(3) Departmental committees reviewed and approved the guidelines
February 2013	The guidelines were used for the first time in the Department
August 2013	Task Force reviewed the guidelines, aligned them with newly published articles, and cited references in the guidelines
December 2013	The revised guidelines were approved by departmental committees
January 2014	Institutional committee approved guidelines for posting on the institution’s intranet
March 2014	(1) Institution’s Legal Department gave its second opinion on the guidelines with suggested editorial changes and gave its approval
	(2) The guidelines were posted on the institution’s intranet

**Table 2:** Initial and updated anticoagulation medication lists.

Initial (October 2012)	Updated (March 2014)	Additions after March 2014
Aspirin	Aspirin and NSAIDs	
Warfarin	Warfarin	
Enoxaparin	LMWH (e.g., enoxaparin or dalteparin SC)	
Heparin	Heparin (IV or SC)	
**Antiplatelet agents**	**Antiplatelet agents**	
Clopidogrel	Clopidogrel (PO)	
Cicagrelor	Cicagrelor (PO)	
Prasugrel	Prasugrel (PO)	
Ticlopidine ^a^	Ticlopidine^a^ (PO)	
	**Phosphodiesterase Inhibitors**	
	Cilostazol (PO)	
**Factor X inhibitors**	**Factor X inhibitors**	**Factor X inhibitors**
Rivaroxaban	Rivaroxaban (PO)	Edoxaban (PO)
	Apixaban (PO)	
	Fondaparinux (SC)	
**Thrombin inhibitors**	**Thrombin inhibitors**	**Thrombin inhibitors**
Dabigatran	Dabigatran (PO)	Desirudin (IV)
	Desirudin (SC)	
	Bivalirudin (IV)	
	Argatroban (IV)	
		**Glycoprotein platelet inhibitors**
		Eptifibatide (IV)
		Tirofiban (IV)
		Abciximab (IV)

Abbreviations: IV, intravenously; LMWH, low-molecular-weight heparin; NSAID, non-steroidal anti-inflammatory drug; PO, orally; SC, subcutaneously; ^a^ Removed from list because drug is no longer marketed.

**Table 3:** Radiology anticoagulation guidelines ^a^.

Feature	Guideline	Comments
**Critical Lab Results**	INR > 1.5 or platelets < 50,000. Notify Radiologist.If high risk of a thromboembolic event off anticoagulation, contact provider for possible bridging therapy.	Procedures with very low bleeding risk, e.g., paracentesis, thoracentesis, thyroid and lymph node biopsy, are not intended to be restricted based on these guidelines and may be performed based on clinical necessity and physician judgment. Notify Radiologist if INR > 3 or platelets < 25,000.
**Agent**	**Recommended Interval between Last Dose and Procedure**	**Recommended Time to Resume Dose**
**Antiplatelet Agents**		
Aspirin and NSAIDS	ASA/dipyridamole: Hold for seven days–preferred. NSAIDS: Hold for five days–preferred. If variance, ok to proceed without Radiologist call for: Abdominal/Ultrasound MSK Neuro Breast Imaging Ultrasound guided core biopsy If variance, notify Radiologist for: Lung nodule biopsies Stereotactic guided breast biopsies MR guided breast biopsies	May resume in 24 h.
Clopidogrel (Plavix) (PO) ticagrelor (Brilinta) (PO)	Hold for five days. Refer back to ordering service with variance.	May resume 24 h post procedure.
Prasugrel (Efficient) (PO)	Hold for seven days. Refer back to ordering service with variance.	Resume as specified by provider.
**Vitamin K Antagonists**		
Warfarin (Coumadin) (PO)	Hold for 3–5 days. Need PT/INR prior to procedure. Notify Radiologist with variance. If variance, ok to proceed with MSK joint procedures	May resume the evening after the procedure.
**Heparins**		
Heparin (IV)	Hold for 4–6 h. No need to check PTT. Notify Radiologist with variance.	May resume 24 h post procedure.
Heparin (SQ)	No need to hold with dose < 10,000 u/day Notify Radiologist with variance.	
Low Molecular Weight Heparin (LMWH), e.g., Enoxaparin/Lovenox and Dalteparin (SQ)	Hold for 24 h (recommend last dose 50% of initial) Under urgent situations literature supports holding for 12 h if eGFR ≥ 45. Notify Radiologist with variance.	May resume 24 h post procedure.
**Phosphodiesterase Inhibitors**		
Cilostazol (Pletal) (PO)	Hold for two days. Refer back to ordering service with variance.	Resume as specified by provider.
**Factor Xa Inhibitors**		
Rivaroxaban (Xarelto) (PO)	If CrCl ≥ 60 mL/min, hold dose two days. If CrCl ≥ 30–59 mL/min, hold dose three days. If CrCl < 15–29 mL/min, hold dose four days. Refer back to ordering service with variance.	May resume 48 h post procedure.
Fondaparinux (Arixtra) (SQ)	Hold for 36–48 h. Refer back to ordering service with variance.	Resume as specified by provider.
Apixaban (Eliquis) (PO) edoxaban (Savaysa) (PO)	If CrCl > 60 mL/min, hold dose 1–2 days. If CrCl 50–59 mL/min, hold dose three days. If CrCl < 30–49 mL/min, hold dose five days. Refer back to ordering service with variance.	May resume 48 h post procedure.
**Thrombin Inhibitors**		
Dabigatran (Pradaxa) (PO)	Hold dose 2–3 days. If CrCl ≥ 50 mL/min, hold dose 2–3 days. If CrCl < 50 mL/min, hold dose 3–5 days. PTT may provide approximation of anticoagulant activity. Refer back to ordering service with variances.	May resume 48 h post procedure.
Desirudin (Iprivask) (SQ)	Hold for 10 h. Refer back to ordering service with variance.	Resume as specified by provider.
Desirudin (Iprivask) (IV)	Hold for 2 h. Refer back to ordering service with variance.	Resume as specified by provider.
Bivalirudin (Angiomax) (IV)	Coagulation times return to baseline approximately 1 h following cessation of drug.	Resume as specified by provider.
Argatroban (IV)	Half-life ranges between 39 and 51 min.	Resume as specified by provider.
**Glycoprotein Platelet Inhibitors**		
Eptifibatide (Integrilin) (IV) Tirofiban (Aggrastat) (IV) Abciximab (ReoPro) (IV)	Eptifibatide and Tirofiban: platelet function returns to baseline 4–8 h after discontinuation. Abciximab: abnormal platelet function for up to seven days after discontinuation.	Resume as specified by provider.

Abbreviations: ASA, aspirin; CrCl, creatinine clearance; eGFR, estimated glomerular filtration rate; INR, international normalized ratio; IV, intravenously; LMWH, low-molecular-weight heparin; NSAID, nonsteroidal anti-inflammatory drug; PO, orally; PTT, partial thromboplastin time; SC, subcutaneously; ^a^ These guidelines are suggested practice guidelines developed by our institution’s Department of Radiology for use by our institution’s Department of Radiology. These guidelines are intended to be used in conjunction with a provider’s training and expertise, as appropriate for the specific assessment and care needs of the individual patient. These practice guidelines are not necessarily inclusive of all proper methods of care, nor are they exclusive of other reasonable methods of care. These guidelines are based on relevant portions of various peer-reviewed publications [4,7,9,10,13] and on the knowledge and expertise of our institution’s Department of Radiology medical staff members. These guidelines represent a consensus among our institution’s Department of Radiology providers to facilitate a consistent, high-quality practice in anticoagulation therapy related to radiology procedures. These guidelines were last updated in January 2017.

## Abbreviations

CrCl	Creatinine clearance
CT	Computed tomographic
IR	Interventional radiology
SIR	Society of Interventional Radiology
US	Ultrasound
